# The Impact of Breast Clinic on the Mastectomy and Axillary Clearance Rates at a Tertiary Hospital in an Eastern Caribbean Nation: A Comparative Study

**DOI:** 10.1155/2019/8018242

**Published:** 2019-03-07

**Authors:** Shariful Islam, Imran Aziz, Jitendra Shah, Jacob Oba, Patrick Harnarayan, Arlene Jammie Rampersad, Vijay Naraynsingh

**Affiliations:** ^1^San Fernando Teaching Hospital, San Fernando, Trinidad And Tobago; ^2^Department of Clinical Surgical Science, University of the West Indies, St. Augustine, Trinidad And Tobago

## Abstract

**Background:**

Breast cancer is the leading form of cancer in women in Trinidad and Tobago. Traditionally the practice of mastectomy or wide local excision with or without axillary clearance was applied to most of these patients. This is often associated with significant morbidity and a poor cosmetic outcome with both negatively impacting the patients quality of life. The aim of our study was to assess the mastectomy and axillary clearance rate before and after the introduction of a specialty breast clinic in September 2012.

**Design and Methods:**

This is a retrospective comparative study of all female patients who underwent breast cancer surgery at our tertiary hospital 3 years prior to and 3 years after starting of breast clinic (between January 2010 and December 2015). Patients were identified from the surgical log books of our hospital. There are 5 surgical units at our hospital and in one of those units the lead surgeon had a special interest in surgical oncoplastic breast surgery. That unit formed the breast clinic in August 2012.

**Results:**

There were 532 women (256 before breast clinic and 276 after breast clinic era) with histologically verified breast cancer operated on between January 2010 and December 2015. The overall mastectomy rate was reduced from 62% to 51% (0.7 to 0.4) and the axillary clearance rate from 66.79% versus 37.31% (0.6 to 0.4) after the introduction of the clinic with p values of 0.007 and 0.009, respectively.

**Conclusions:**

The introduction of breast clinic has significantly reduced the mastectomy and axillary clearance rate at our teaching hospital.

## 1. Introduction

Breast cancer is the leading form of cancer in women in Trinidad and Tobago and also the commonest cause of death from cancer among women worldwide and in Trinidad and Tobago. Trinidad and Tobago is an Eastern Caribbean country with the highest breast cancer mortality in the Caribbean; 45% of women were diagnosed at stage 1 or 2 [1]. Traditionally the practice of mastectomy or wide local excision with or without axillary clearance was applied to most of these patients and, until today, most of these surgeries are done by the general surgeons. This method of treatment is often associated with significant morbidity and it creates great distress and fear amongst the female population with a poor cosmetic outcome which negatively impact patient's quality of life. Cosmesis is very important to these patients and there is now growing demand for more and more cosmetically pleasing surgeries. To meet this demand, surgeons are now required to have further training or specializing in the field of breast cancer surgery. Specialization helps the surgeons to gain in-depth knowledge and a better understanding of the breast cancer management and also helps them acquire special skill sets, i.e., different oncoplastic breast surgeries for the management of breast cancer patients. Because of this reason, special breast unit has now been established in many hospitals around the world.

Worldwide the mastectomy rate has dropped over the years. In USA the mastectomy rate was 77% in 1988 and it was reduced to 38% in 2004. Similarly in Europe in 2005 the rate was 29.9% and 18.6% in 2010 [1]. Additionally the Axillary lymph node dissections (ALND) were also slowly fading into the background. At the same time, BCS was established as a safe option for most women with early breast cancer. In fact, the 5-year survival of BCS with radiation is not statistically different when compared with mastectomy alone in patients with stage I or II breast cancers. We compared the mastectomy and axillary clearance rate in the pre- and post-breast clinic era between January 2010 and December 2015 at our tertiary hospital to investigate what impact the Breast clinic had on the mastectomy rate and ALND rate.

## 2. Study Design and Method

It is a retrospective comparative study performed at our tertiary teaching hospital between January 2012 and December 2015. The period was divided into pre-breast clinic era (January 2010 to August 2012) and post-breast clinic era (September 2012 to December 2015). Approval was obtained from the institutional review board to conduct this study.

### 2.1. Inclusion Criteria

All patients who underwent surgical intervention to the breast related to breast cancer or carcinoma in situ during this period were included into this study.

### 2.2. Exclusion Criteria

There were no exclusion criteria.

### 2.3. Data Extraction

Permission was obtained from all consultants who treated the breast cancer patients during this period. Data was obtained from the surgical log books and samples were divided to “pre breast clinic” and “post breast clinic” group. Information on patients included demographics (age, sex), time of surgery (pre- or post-breast clinic era), and type of surgery performed.

### 2.4. Statistical Analysis

Data was entered into Microsoft Excel spread sheet and analysed using SPSS version 15.0. The mastectomy rate between pre- and post-breast clinic era was calculated and chi-square test performed to determine the statistical significance. Similarly, the axillary clearance rate was also calculated between the two periods and Fisher exact test was performed to determine the statistical significance. A p value of .005 was set to be statistically significant.

## 3. Results

A total of 532 patients underwent breast cancer surgery during our study period by the all the surgical units including the breast unit, of which 256 (48.12%) breast cancer surgeries were performed in the pre-breast clinic period compared to 276 (51.87%) in the post-breast clinic period (see Table 1). 99.06% of our patients (n=527) were females and only 0.94% (n=5) were males.

### 3.1. Demographics

**Figure 1:**
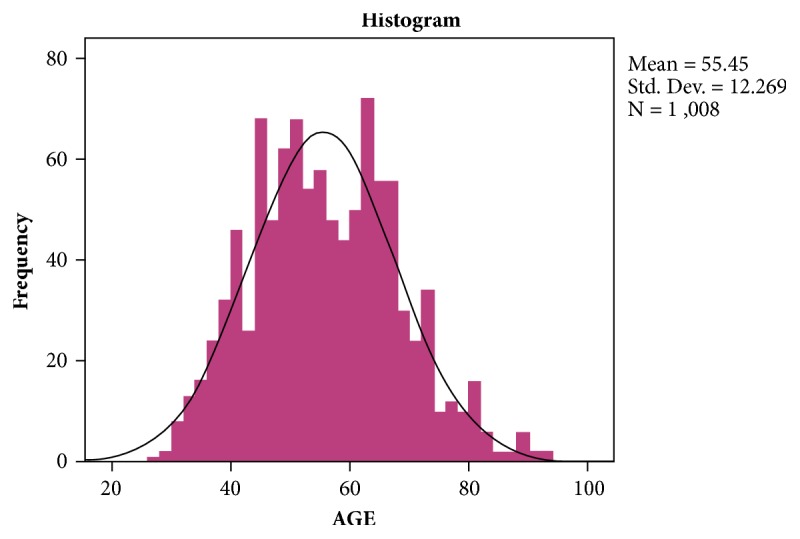
Distribution of the age of the patients in all the surgical units (mean and median 55, mode 44, and range 27-92).

The mean and the median age of our patients were 55 with a mode of 44. The age groups ranged from the minimum of 27 years to the maximum of 92 years. The frequency distributions of the patient's age were also shown on the histogram (see Figure 1).

### 3.2. Distribution of Patients among the Different Surgical Units

**Figure 2:**
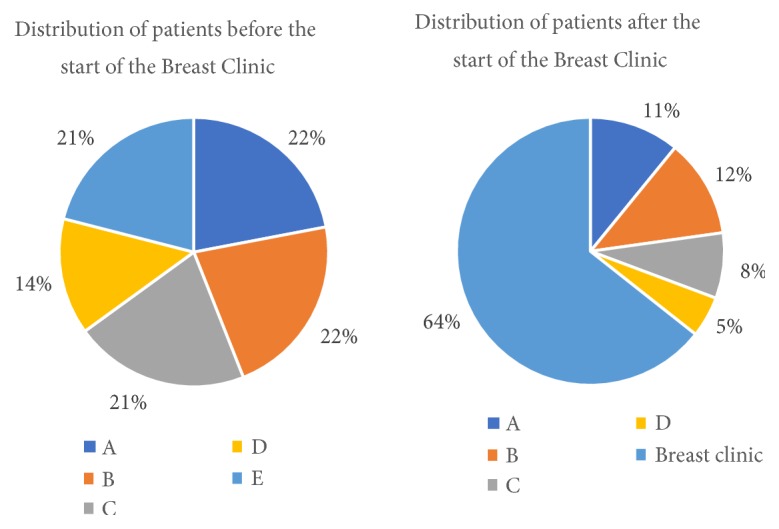
Distribution of patients among all the surgical units.

Before the commencement of the breast clinic, the number of surgeries performed by each surgical unit was more or less evenly distributed amongst the surgical units with one exception. Although all the surgical units continued to perform the breast cancer surgery even after starting of the breast clinic; it was noted that there was a dramatic shift in the volume of patients towards the breast unit from the pre-breast clinic period 22% to the post-breast clinic period 65% (see Figure 2).

### 3.3. The Mastectomy Rate

The mastectomy rate in all the surgical units in the pre-breast clinic period was 62.5% whereas in post-breast clinic period it was 51%. This reduction of mastectomy rate in the post-breast clinic period was due to corresponding rise of wide local excision rate from 37.5% to 48.91%, as more and more patients underwent breast conserving surgery during this period (see Table 2).

**Figure 3:**
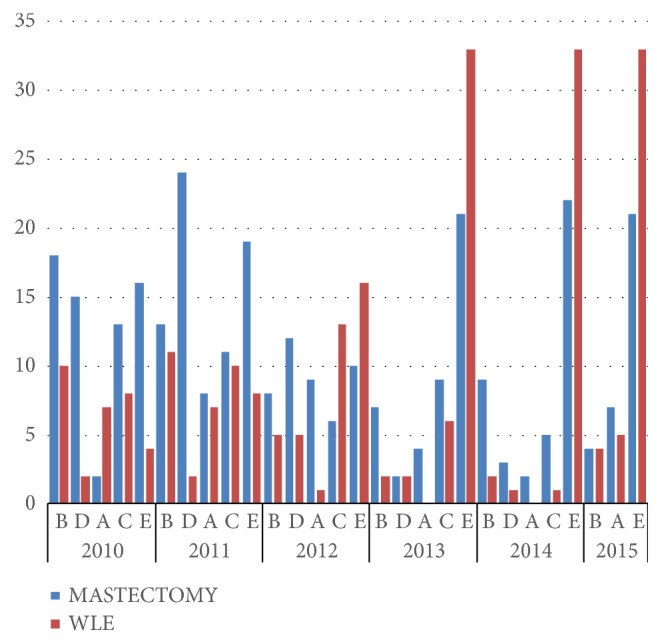
Pattern of mastectomy rates over the years in all the surgical units. Note that unit E evolved into the breast unit mid 2012.

There was a significant decrease in the cumulative mastectomy rate from 0.62 to 0.4 in the post-breast clinic period when compared with those in the pre-breast clinic period (see Figure 3). This reduction of mastectomy rate in post-breast clinic period with corresponding rise of WLE rate was not only limited to the breast clinic alone but also extends to all other surgical units as well. A chi-square test was performed to find out whether this finding was statistically significant or not. The statistic was 7.0418 and the p-value was less than .007963.

### 3.4. The Axillary Clearance Rate

**Figure 4:**
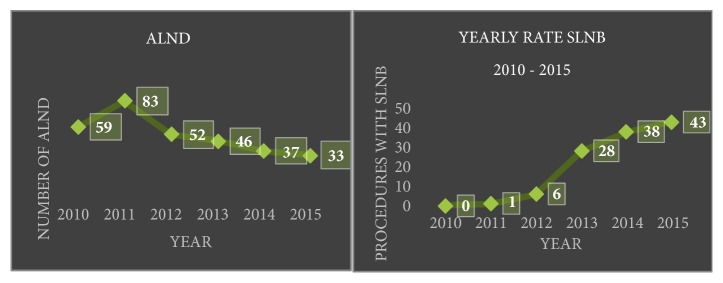
Pattern of axillary clearance rate and sentinel lymph node biopsy rate over the years in all the surgical units.

With the introduction of the sentinel lymph node biopsy technique for axillary staging in 2011; there was a linear regression in the numbers of patients who underwent complete axillary clearance. The total number of patients that underwent axillary clearance rate in the pre-breast clinic period was 171 out of 256 (66.79%) versus 103 out of 276 (37.31%) in the post-breast clinic period (Table 3) and this was because of corresponding rise in sentinel lymph node biopsy technique during the same period of time (Figure 4). The difference in overall rate of axillary clearance was statistically different from the breast clinic. This can be explained by rise in the number of SLNB performed in the breast clinic cohort.

## 4. Discussion

Trinidad and Tobago is an Eastern Caribbean country with the highest breast cancer mortality in the Caribbean; 45% of women were diagnosed at stage 1 or 2 [2]. The ethnic composition of this country is as follows: 45.9% of the population are of African descent, 27.5% are of Indian descent, 14.7 % are of mixed ethnicity, and 12 % are of other ethnic backgrounds.

The incidence of breast cancer is increasing in Trinidad and Tobago; approximately 250 new cases are diagnosed each year and 125 deaths per year. More than 33% of the new cases are < 50 years old at diagnosis versus 15-20%. 80% of our cases have stage I and II disease at diagnosis [3]. This indicates the need to introduce specialist service in the management of breast cancer surgery to improve the overall standard of care in these patients.

Breast surgery has become more minimalistic. It is much more complex in decision-making. Cosmesis is now very important which helps patient to recover psychologically as well as physically.

Treating breast cancer almost always involves surgery and for years the choice was lumpectomy and or mastectomy. The newer breast conserving approaches have dramatically changed this field surgery giving women more options, fewer long-term side effects, smaller scars, and better cosmetic results.

Breast conservation surgery (BCS) has been established as a safe option for most women with early breast cancer. In fact, the 5-year survival of BCS with radiation is not statistically different when compared with mastectomy alone in patients with stage I or II breast cancer [4, 5].

BCS has become the standard of care of early breast cancer and survival is now excellent. However the focus of BCS has now shifted to cosmetic outcome, quality of life, and patient's satisfaction. Nonetheless, excision of certain tumours still presents a considerable challenge.

In 1996 Audretsch et al. introduced the new technique of oncoplastic breast surgery. This specialized breast surgery utilizes plastic surgical techniques without jeopardizing oncological principles [6]. It provides a wider local excision while still achieving the goals of a better breast shape and symmetry [7].

To meet this growing demands, surgeons are now required to obtain further specialized training in this filed to keep them up to date. Based on this background specialized breast service or breast unit has been established in many hospitals around the world. However, to establish an effective breast unit, certain things need to be in place, i.e., adequately trained personnel, facilities for proper investigation, reputable and timely available pathology service, timely radiation and chemotherapy, adequate social and psychological support system, and a multidisciplinary and patient centred approach.

Despite this development in the management of breast cancer patients, specialized breast service did not start formally in the public hospital of Trinidad and Tobago until September 2012, when a general surgeon with special interest in breast started this clinic. However it was a not an easy task as the general surgeons often feel that it is not necessary to start this specialized breast clinic as they think that others can offer the same type of service with similar outcomes.

Contrary to this claims several studies have documented that treatment done by specialized unit is far more superior to nonspecialized unit. This outcome measure is not only limited to the quality of care or extent of surgery but also translated to a decrease in recurrence rate as well as improving overall survival of the patients. Initially two major observational studies have reported this survival benefit [8, 9]. However, there are limitations in these studies as neither of these studies could determine the exact treatment offered and its adequacy nor can it critically assess the overall impact on locoregional recurrence and survival.

The landmark study by D Kingsmore et al. in 2004 effectively answers many of these questions. They compared the adequacy of surgical management, local recurrence rates, and the survival outcomes of specialists and nonspecialists in 2148 breast cancer patients over an 8-year follow-up period. This study noted that patients treated by the specialist unit had a 50% reduction in the risk of inadequate treatment of the breast, a nine times lower risk of inadequate definitive axillary treatment, and fivefold lower risk of inadequate axillary staging compared to the nonspecialist unit (24 vs 47%, Po0.001), 4 vs 38%, Po0.001) and (8 vs 40%, Po0.001) respectively. The local recurrence rates were 57% lower (13 versus 23% at eight years, Po0.001) in patients treated by the specialist [10].

The adequacy of the surgical treatment is the key factor to the improvement in overall survival of breast cancer patients. Surgical management is more often adequate with a lower locoregional recurrence rates in specialized breast units compared to the nonspecialized unit. Most importantly D Kingsmore et al.'s study noted that the risk of death from breast cancer was 20% lower for patients treated in specialist units [10].

The factor of recording adequate clinical and complete histopathological data is of paramount importance in the management of breast cancer patients. As this will prevent many patients from getting the best available therapy and thereby negatively affects their outcomes as well as overall survival. Once again D Kingsmore et al.'s study noted that there are many flaws in the management of breast cancer patients by the nonspecialized unit compared to the specialized unit. Most of their pathological data is incomplete. Exact tumour size (8 versus 24%), grade (20 versus 61%), oestrogen receptor status (18 versus 38%), and margin status (reported only in 58% of women with conservation surgery) are often unknown. It was also noted that there is frequent omission of axillary staging (7 versus 21%) as well as axillary nodal status. Unfortunately, even a preoperative histological diagnosis was less often made by the nonspecialists (81 versus 33%, Po0.001) [10]. Therefore the exact pathological stage was less completely reported in nonspecialist units.

Not only that, nonspecialist surgeons frequently leave positive margins after excision (4 versus 12%, Po0.01) and omitted radiotherapy more often (16 versus 31%, Po0.01). Contrary to this, specialist surgeons omitted radiotherapy after conservation surgery more selectively for tumours with an excellent prognosis [10].

These missed pieces of information lead to undertreatment or inappropriate treatment of these cancer patients which in turn increases the risk of future locoregional recurrence and thereby decrease overall survival [10] which is also consistent with the findings of a recently published randomized trials of postmastectomy radiotherapy in high-risk women [11, 12].

Last but not least, overall patient's survival depends on the workload of the surgeons performing the surgery. Patients treated by a low-workload surgeon had a poorer five-year survival compared to a high-workload surgeon, 60% versus 68%. Comparing the surgeons with high workload (>50 per annum) to surgeons with workloads <10 and 10-29 cases per annum, the relative risk of death was increased by 15% and 10%, respectively. This study provides further solid evidence that the management of patients by surgeons with low workloads decreases the overall survival. Therefore the authors recommended that general surgeons should obtain specialized training in this filed for better management of their breast cancer patients [13].

Compared to all the above studies our study is limited to many aspects of the management. Our study was not fully designed to look at the detailed management and outcome difference between the two groups. The only aim of our study was limited to the mastectomy and axillary clearance rate between the pre- and post-breast clinic period. However a future study incorporating the above parameters can in fact answer many of the above questions.

Despite all of our shortcomings, our study has shown that the breast clinic has successfully reduced the mastectomy and axillary clearance rate in our breast cancer patients. The other important thing to note from our study is that this overall reduction of mastectomy and axillary clearance rate happened even though 45% of our breast cancer surgery in the post-breast clinic area was performed by the nonspecialist surgeons. This finding further signifies the positive impact of the breast clinic on the overall management of breast cancer patients even among nonspecialist surgeons.

The role of reconstructive surgeons in the management of these breast cancer patients is very important. Because of their active involvement, surgeons are encouraged to excise a larger lesion without any fear of the oncological safety or cosmetic deformity or having to a do disfiguring mastectomy. Similarly surgeons are also gaining tips from the reconstructive surgeons to do the majority of their breast surgery. In future, the breast surgeons with further specialization will be able to manage these patients with adequate oncological safety as well as good cosmetic outcome.

## 5. Conclusion

The benefit of specialist care was apparent from our study. Our results have shown that the introduction of breast clinic had significantly reduced the mastectomy rate as well as axillary clearance rate at our teaching hospital, which further emphasizes the need for specialized service in any aspect of surgery to improve the overall standard of care for the cancer patients. Despite all of the barricades for establishing any specialized service at any institute by the general surgeons; its efficacy in the outcomes of patient management is unparalleled. Although our rates are approaching first-world levels however more work is needed. More and more surgeons are needed to be trained in this specialized filed to improve the patient care and to improve the overall survival of these breast cancer patients. The oncoplastic breast surgery can be effectively applied in the treatment of early breast cancers in our patients without the need of unnecessary mastectomy, without jeopardizing the risk of further morbidity and mortality.

### 5.1. Limitation

It is retrospective study and also the study period is short as the breast clinic started in our hospital only few years ago. The numbers of patients are small, as the population of our country is only 1.3 million and our hospital served only 400-500,000 populations. A future prospective study will be conducted in future to document the cosmetic outcomes, psychological impact, and recurrence as well as survival rates between these two groups.

## Figures and Tables

**Table 1:** Number of patients in the pre- and post-breast clinic period in all the surgical units.

Time series	Total no. of patients
Before breast clinic (January 2010-August 2012)	256
After breast clinic (September 2012 to December 2015)	276
Total	532

**Table 2:** The percentage of Mastectomy and WLE in the pre- and post-breast clinic period.

Time series	Mastectomy	WLE	Total	Rate
Before breast clinic	160	96	256	62%
After breast clinic	141	135	276	51%

**Table 3:** Percentage of axillary clearance rate in pre- and post-breast clinic period in all the surgical units.

Time period	Axillary clearance	No axillary clearance	Total	Rate
Before breast clinic	171	85	256	66.79%
After breast clinic	103	173	276	37.31%

## Data Availability

The data used to support the findings of this study are available from the corresponding author upon request.

## Additional Points

*Highlights*. (1) Management of any cancer patient requires a holistic approach and it can only be achieved by a specialist surgeon rather than by a general surgeon. (2) The benefits of specialist care are now well proven; therefore the care by the nonspecialist is now slowly fading away. (3) Specialist management not only improves the quality of care but also improves the overall patient's survival. (4) Breast clinic through its specialized service can effectively offer this care to all the breast cancer patients.
